# *catena*-Poly[[chloridodimethyl­tin(IV)]-μ-chloro­acetato-κ^2^ *O*:*O*′]

**DOI:** 10.1107/S160053681104918X

**Published:** 2011-11-23

**Authors:** Shaoliang Zhang, Junhong Zhang, Rufen Zhang

**Affiliations:** a College of Chemistry and Chemical Engineering, Liaocheng University, Shandong 252059, People’s Republic of China

## Abstract

In the title polymeric coordination compound, [Sn(CH_3_)_2_(C_2_H_2_ClO_2_)Cl]_*n*_, the Sn atom has a distorted trigonal–bipyramidal geometry, with two O atoms of the ligands in axial positions and two methyl groups and one Cl atom in equatorial positions. Adjacent Sn atoms are bridged by the two O atoms of the carboxylate ligand, forming a chain structure along the *a*-axis direction.

## Related literature

For the biological activity of organotin compounds, see: Dubey & Roy (2003 ▶). For related structures, see: Wang *et al.* (2007 ▶); Ma *et al.* (2008 ▶).
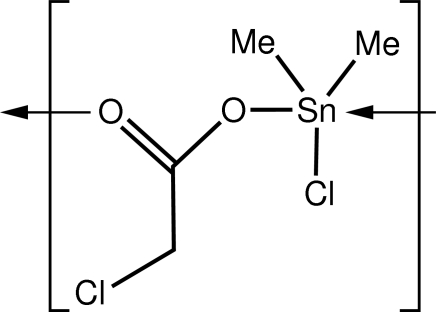


## Experimental

### 

#### Crystal data

[Sn(CH_3_)_2_(C_2_H_2_ClO_2_)Cl]
*M* *_r_* = 277.69
Monoclinic, 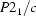

*a* = 6.988 (3) Å
*b* = 9.948 (4) Å
*c* = 12.686 (6) Å
β = 98.891 (5)°
*V* = 871.3 (7) Å^3^
*Z* = 4
Mo *K*α radiation
μ = 3.48 mm^−1^
*T* = 298 K
0.39 × 0.38 × 0.15 mm

#### Data collection

Siemens SMART CCD area-detector diffractometer
Absorption correction: multi-scan (*SADABS*; Sheldrick, 1996) ▶ *T*_min_ = 0.344, *T*_max_ = 0.623
4254 measured reflections
1527 independent reflections
1145 reflections with *I* > 2σ(*I*)
*R*_int_ = 0.057

#### Refinement

*R*[*F* ^2^ > 2σ(*F* ^2^)] = 0.040
*wR*(*F* ^2^) = 0.100
*S* = 1.08
1527 reflections
84 parameters
H-atom parameters constrained
Δρ_max_ = 0.76 e Å^−3^
Δρ_min_ = −0.85 e Å^−3^

### 

Data collection: *SMART* (Siemens, 1996 ▶); cell refinement: *SAINT* (Siemens, 1996 ▶); data reduction: *SAINT*; program(s) used to solve structure: *SHELXS97* (Sheldrick, 2008 ▶); program(s) used to refine structure: *SHELXL97* (Sheldrick, 2008 ▶); molecular graphics: *SHELXTL* (Sheldrick, 2008 ▶); software used to prepare material for publication: *SHELXTL*.

## Supplementary Material

Crystal structure: contains datablock(s) I, global. DOI: 10.1107/S160053681104918X/kp2369sup1.cif

Structure factors: contains datablock(s) I. DOI: 10.1107/S160053681104918X/kp2369Isup2.hkl

Additional supplementary materials:  crystallographic information; 3D view; checkCIF report

## Figures and Tables

**Table 1:** Selected bond lengths (Å)

Sn1—C3	2.089 (6)
Sn1—C4	2.100 (7)
Sn1—O1	2.152 (4)
Sn1—Cl2	2.352 (2)
Sn1—O2^i^	2.493 (5)

Symmetry code: (i) 
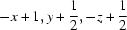
.

**Fig. 1.**
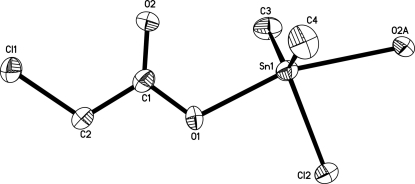
The asymmetric unit of the title compound, showing 30% probability displacement ellipsoids. H atoms have been omitted for clarity.

**Fig. 2.**
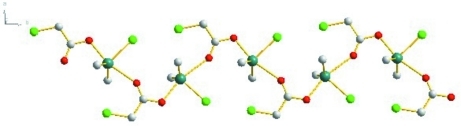
A view of the polymeric structure of the title compound.

## supplementary crystallographic information

### Comment

In recent years, organotin compounds have been attracting more and more attention due to their wide range of industrial applications and biological activities (Dubey & Roy, 2003). As a part of our ongoing investigations in this field, we have synthesised the title compound and present its crystal structure here. The title compound (Fig. 1) forms an extended chain arising from Sn—O bridges formed by the chloroacetic acid ligands. The Sn—O bond distances in the compound (Sn1—O1 = 2.152 (4) Å; Sn1—O2 = 2.493 (5) Å;) are comparable to those found in a related organotin carboxylate (Ma *et al.*, 2008). The Sn atom is five-coordinate in a slightly distorted trigonal-bipyramidal coordination geometry, provided by the methyl groups and chlorine atom in the equatorial positions and the two coordinated O atoms in the axial positions (Table 1).

### Experimental

The reaction was carried out under a nitrogen atmosphere. Chloroacetic acid (1 mmol) and sodium ethoxide (1 mmol) were added to a solution of benzene (30 mL) in a Schlenk flask with continuous stirring for 0.5 h. Then dimethyltin dichloride (1 mmol) was added to the reactor and the reaction mixture was stirred for 12 h at room temperature. The resulting clear solution was evaporated under vacuum. The product was crystallised from a solution of diethyl ether to yield colourless blocks of the title compound (yield 78%). Anal. Calcd (%) for C_4_H_8_Cl_2_O_2_Sn_1_(Mr = 277.69) :*C*, 17.30; H, 2.90. Found (%): C, 17.55; H, 2.62.

### Refinement

The H atoms were positioned geometrically, with methyl C—H distances of 0.96Å and aromatic C—H distances of 0.93 Å, and refined as riding on their parent atoms, with *U*_iso_(H) = 1.2 *U*_eq_(C) or 1.5 *U*_eq_(C) for the methyl groups.

### Crystal data

**Table:**

[Sn(CH_3_)_2_(C_2_H_2_ClO_2_)Cl]	*F*(000) = 528
*M**_r_* = 277.69	*D*_x_ = 2.117 Mg m^−^^3^
Monoclinic, *P*2_1_/*c*	Mo *K*α radiation, λ = 0.71073 Å
Hall symbol: -P 2ybc	Cell parameters from 1496 reflections
*a* = 6.988 (3) Å	θ = 3.0–25.4°
*b* = 9.948 (4) Å	µ = 3.48 mm^−^^1^
*c* = 12.686 (6) Å	*T* = 298 K
β = 98.891 (5)°	Block, colourless
*V* = 871.3 (7) Å^3^	0.39 × 0.38 × 0.15 mm
*Z* = 4	

### Data collection

**Table:**

Siemens SMART CCD area-detector diffractometer	1527 independent reflections
Radiation source: fine-focus sealed tube	1145 reflections with *I* > 2σ(*I*)
graphite	*R*_int_ = 0.057
phi and ω scans	θ_max_ = 25.0°, θ_min_ = 2.6°
Absorption correction: multi-scan (*SADABS*; Sheldrick, 1996)	*h* = −8→8
*T*_min_ = 0.344, *T*_max_ = 0.623	*k* = −10→11
4254 measured reflections	*l* = −13→15

### Refinement

**Table:**

Refinement on *F*^2^	Primary atom site location: structure-invariant direct methods
Least-squares matrix: full	Secondary atom site location: difference Fourier map
*R*[*F*^2^ > 2σ(*F*^2^)] = 0.040	Hydrogen site location: inferred from neighbouring sites
*wR*(*F*^2^) = 0.100	H-atom parameters constrained
*S* = 1.08	*w* = 1/[σ^2^(*F*_o_^2^) + (0.0422*P*)^2^] where *P* = (*F*_o_^2^ + 2*F*_c_^2^)/3
1527 reflections	(Δ/σ)_max_ = 0.006
84 parameters	Δρ_max_ = 0.76 e Å^−^^3^
0 restraints	Δρ_min_ = −0.85 e Å^−^^3^

### Special details

**Table:**

Geometry. All e.s.d.'s (except the e.s.d. in the dihedral angle between two l.s. planes) are estimated using the full covariance matrix. The cell e.s.d.'s are taken into account individually in the estimation of e.s.d.'s in distances, angles and torsion angles; correlations between e.s.d.'s in cell parameters are only used when they are defined by crystal symmetry. An approximate (isotropic) treatment of cell e.s.d.'s is used for estimating e.s.d.'s involving l.s. planes.
Refinement. Refinement of *F*^2^ against ALL reflections. The weighted *R*-factor *wR* and goodness of fit *S* are based on *F*^2^, conventional *R*-factors *R* are based on *F*, with *F* set to zero for negative *F*^2^. The threshold expression of *F*^2^ > σ(*F*^2^) is used only for calculating *R*-factors(gt) *etc*. and is not relevant to the choice of reflections for refinement. *R*-factors based on *F*^2^ are statistically about twice as large as those based on *F*, and *R*-factors based on ALL data will be even larger.

### Fractional atomic coordinates and isotropic or equivalent isotropic displacement parameters (Å^2^)

**Table:**

	*x*	*y*	*z*	*U*_iso_*/*U*_eq_	
Sn1	0.57933 (7)	0.15030 (4)	0.27295 (3)	0.0368 (2)	
Cl1	0.8748 (3)	−0.36021 (16)	0.41225 (19)	0.0634 (6)	
Cl2	0.8014 (3)	0.32389 (17)	0.32699 (17)	0.0570 (5)	
O1	0.7885 (7)	0.0210 (4)	0.3651 (4)	0.0494 (13)	
O2	0.6022 (7)	−0.1521 (4)	0.3121 (4)	0.0491 (13)	
C1	0.7549 (11)	−0.1063 (6)	0.3580 (5)	0.0376 (16)	
C2	0.9233 (11)	−0.1869 (6)	0.4116 (6)	0.0480 (19)	
H2A	0.9576	−0.1560	0.4846	0.058*	
H2B	1.0337	−0.1714	0.3753	0.058*	
C3	0.6184 (11)	0.0809 (7)	0.1223 (5)	0.052 (2)	
H3A	0.5372	0.0040	0.1036	0.078*	
H3B	0.7515	0.0561	0.1235	0.078*	
H3C	0.5844	0.1507	0.0705	0.078*	
C4	0.3673 (12)	0.1411 (7)	0.3734 (6)	0.058 (2)	
H4A	0.3176	0.2296	0.3823	0.087*	
H4B	0.4237	0.1059	0.4416	0.087*	
H4C	0.2637	0.0834	0.3421	0.087*	

### Atomic displacement parameters (Å^2^)

**Table:**

	*U*^11^	*U*^22^	*U*^33^	*U*^12^	*U*^13^	*U*^23^
Sn1	0.0406 (4)	0.0330 (3)	0.0351 (3)	−0.0031 (2)	0.0000 (2)	−0.0039 (2)
Cl1	0.0525 (14)	0.0306 (10)	0.0986 (17)	0.0054 (8)	−0.0153 (11)	0.0021 (9)
Cl2	0.0574 (14)	0.0351 (9)	0.0700 (13)	−0.0061 (8)	−0.0164 (10)	−0.0051 (9)
O1	0.056 (3)	0.028 (2)	0.056 (3)	0.007 (2)	−0.016 (2)	0.007 (2)
O2	0.041 (3)	0.039 (3)	0.060 (3)	−0.002 (2)	−0.018 (2)	−0.002 (2)
C1	0.044 (5)	0.031 (4)	0.038 (4)	−0.002 (3)	0.008 (3)	−0.004 (3)
C2	0.049 (5)	0.029 (4)	0.061 (5)	−0.004 (3)	−0.006 (4)	−0.002 (3)
C3	0.053 (5)	0.065 (5)	0.039 (4)	−0.007 (4)	0.011 (3)	−0.016 (4)
C4	0.059 (6)	0.066 (5)	0.052 (5)	0.009 (4)	0.017 (4)	0.005 (4)

### Geometric parameters (Å, °)

**Table:**

Sn1—C3	2.089 (6)	C1—C2	1.498 (10)
Sn1—C4	2.100 (7)	C2—H2A	0.9700
Sn1—O1	2.152 (4)	C2—H2B	0.9700
Sn1—Cl2	2.352 (2)	C3—H3A	0.9600
Sn1—O2^i^	2.493 (5)	C3—H3B	0.9600
Cl1—C2	1.757 (6)	C3—H3C	0.9600
O1—C1	1.289 (7)	C4—H4A	0.9600
O2—C1	1.221 (8)	C4—H4B	0.9600
O2—Sn1^ii^	2.493 (5)	C4—H4C	0.9600
			
C3—Sn1—C4	138.1 (3)	Cl1—C2—H2A	109.0
C3—Sn1—O1	97.2 (2)	C1—C2—H2B	109.0
C4—Sn1—O1	97.4 (3)	Cl1—C2—H2B	109.0
C3—Sn1—Cl2	109.6 (2)	H2A—C2—H2B	107.8
C4—Sn1—Cl2	110.5 (2)	Sn1—C3—H3A	109.5
O1—Sn1—Cl2	85.32 (13)	Sn1—C3—H3B	109.5
C3—Sn1—O2^i^	89.7 (2)	H3A—C3—H3B	109.5
C4—Sn1—O2^i^	86.4 (2)	Sn1—C3—H3C	109.5
O1—Sn1—O2^i^	164.60 (16)	H3A—C3—H3C	109.5
Cl2—Sn1—O2^i^	79.39 (13)	H3B—C3—H3C	109.5
C1—O1—Sn1	116.6 (5)	Sn1—C4—H4A	109.5
C1—O2—Sn1^ii^	148.1 (4)	Sn1—C4—H4B	109.5
O2—C1—O1	122.4 (7)	H4A—C4—H4B	109.5
O2—C1—C2	125.7 (6)	Sn1—C4—H4C	109.5
O1—C1—C2	111.8 (6)	H4A—C4—H4C	109.5
C1—C2—Cl1	112.9 (5)	H4B—C4—H4C	109.5
C1—C2—H2A	109.0		

Symmetry codes: (i) −*x*+1, *y*+1/2, −*z*+1/2; (ii) −*x*+1, *y*−1/2, −*z*+1/2.
